# Mr. Hull's Account of a Case of Hæmoptoe

**Published:** 1800-05

**Authors:** Thomas Hull

**Affiliations:** East Retford


### To the Editors of the Medical and Phyfical Journal.

Gentlemen,

THE liberty I have taking in communicating to the world, through the medium of your valuable Journal, the fubfequent cafe; which, on account of fome few attendant fingularities, feems defervedly to call for a place in the annals of medical literature; I am perfuaded you will readily excufe, when it is confidered, what rapid and profitable ftrides have been made in the practical department of medicine, from a due and proper attention to, and a juft and faithful delineation of the vaft variety of fingularities, incident to almoft all difeafes, and all their ftages.

John Jackfon, aged 21 years, a cabinet-maker, of a fanguine and plethoric ftate of body, ftout and robuft, rather inclined clined to corpulency, not peculiarly ſhort in the neck, nor in any degree contracted in the cheſt, was, on the evening of Feb. 3, ſuddenly ſeized with violent dyſpnœa, which, on motion, or the ſlighteſt attempt to walk, was ſo conſiderably increaſed, as to threaten almoſt immediate ſuffocation. This difficulty of breathing came on about ſix o'clock, P. M. at which time alſo, by the efforts in coughing, and the unuſually violent pulſation of the heart and arteries, a conſiderable diſcharge of blood took place; the quantity, however, though very conſiderable, could not, on account of its admixture with mucus and ſaliva, be accurately aſcertained. About nine o'clock every ſymptom increaſed, and the ſucceſſion of others, ſtill more alarming than the firſt, ſeemed evidently to indicate a rapid advance towards a fatal termination. At half paſt twelve o'clock in the morning, I was deſired to viſit the patient, at which time I found him in ſo deſperate a ſituation, as to preclude the leaſt ray of hope. The pulſation at the wriſt was *frequent*, about 110 in a minute, extremely *full*, *quick*, and perfectly regular as to force, without intermiſſion. The pulſation of the heart was ſo violent, as to be ſeen and diſtinctly heard. The reſpiration was to the pulſation of the heart in the proportion of about 4.25 to 5.5. The blood was occaſionally diſcharged from the lungs, almoſt unconnected with mucus, and thrown to a prodigious diſtance, (if we may uſe the expreſſion) "exultim." This circumſtance, however, did not take place but through the intervention of the cough, and not often, as by far the greater quantity of blood was diſcharged mixed with mucus, "extuſſiendo." An intolerable ſenſe of tightneſs in the cheſt, was the moſt oppreſſive and alarming ſymptom attendant on the complaint. Being laid upon his back, he felt as if tied down by cords, unable to move, or make the leaſt reſiſtance. An acute pain ſtriking from betwixt the ſhoulders to the ſternum, was the firſt ſymptom that gave him any conſiderable uneaſineſs, to which ſucceeded the difficulty of breathing. About midnight the left arm became ſo painful, as to cauſe conſiderable reſtleſsneſs: this ſymptom continued to the laſt. All the excretions were perfectly regular. About two years ago, I was informed, he had been attacked with a ſimilar complaint, though in a milder degree, which was attended by a ſlight diſcharge of blood from the lungs, produced by no known cauſe, and which in the ſpace of a few days he recovered from. Ever ſince that time, however, he has been very liable, from the ſlighteſt cold, or over-exertion, to be affected with what he calls an aſthmatical complaint, and occaſionally to be troubled with hæmoptoe. He can aſſign no cauſe for the preſent attack. From knowing the nature of his occupation, I was induced to think, that that an over-exertion, in this inftance, might have been the caufe; but this, he afferts, was not the cafe. For about a week previous to his death, he has occafionally difcharged a portion of blood from the rectum. His ftrength has been fo much impaired within the laft two months, as to prevent him executing his accuftomed work, though chiefly employed within doors.

Since the firft attack, he has been fenfibly affected by change of weather, efpecially from a moift atmofphere; and conftantly complained of the difficulty of keeping himfelf warm in bed. During the laft nine months, he has fported too much with his conftitution, having fubmitted himfelf to the care of an empiric in the neighbourhood, and ufed feveral quack medicines, with the nature of which I am unacquainted. On the night previous to his death, a confiderable obftruction in the œfophagus took place, fo as to render deglutition extremely difficult.

The patient, though in this defperate and, to all appearance, irrecoverable fituation, was not to be neglected; the ties of duty and humanity call for our utmoft exertions: I ordered that cloths, dipped in cold water, fhould be immediately applied to the fcrobic. cordis, and ufed for a confiderable length of time, which was evidently attended with good effects. In conjunction with the above, I prefcribed the following draught:

℞. Æther vitriolic ʒj. tinct. opii. gt. xx. pulv. caftor rus. gr. vj. aq. nuc. mofchat. ℥j. ft. hauft extemplò fumendus, et poft horas duas, nifi prius remiferint figna, repetendus.

In the fpace of about half an hour, as the hæmorrhage had ftopped and the fymptoms fomewhat abated, and as he feemed in a great meafure inclined to fleep, I left him to the care of his nurfe.

4to. At feven o'clock I vifited him this morning—he had taken both his draughts; but ftill the great difficulty of breathing continued, with the pain of his arm—no further hæmorrhage—had enjoyed no fleep—was perfectly coherent—pulfation at the wrift totally gone; yet he confidered and exprefsed himfelf as much eafier, *affuring* me, that if I could only remove the "cramp at his heart", he fhould be well. The laft and only chance for his life, feemed now to depend upon the poffible good effects from the application of a blifter to the fcrobic. cordis, which was immediately done; at the fame time, I prefcribed him the following, which I believe was never taken:

℞. Mofch. fmens. gr. xv. mift. camphor ʒij. tinct. cinch. flav. ℥fs. aq. nuc. mofch. ℥j. M. ft hauft. ftatim fumendus.

About two o'clock, P. M. he died; and I am forry to fay, I had no opportunity of examining his body after death.

Such

Such being the hiſtory of the caſe, I hope it will not be deemed impertinent if I trouble you with a few comments.

As I had great reaſon to believe, from inquiries previouſly made, that he was of a phthiſical habit of body, and, as aforetime, he had been troubled with many ſymptoms indicative of pulmonary conſumption, it was natural to infer, that either a vomica, or the conſequences of the previous hæmoptoe, added conſiderably to his preſent diſtreſſes; and that through the means of ſuch violent exertions, this incipient vomica might have burſt, or the cicatrix left by the hæmoptoe again opened, which would have accounted for the vaſt diſcharge of blood, through the ſhort courſe of this diſeaſe. From many circumſtances, I am induced to think that this efflux of blood aroſe from the latter cauſe. If a vomica had previouſly been formed in the lungs, and now burſt, it is very evident that from ſuch cauſes, a great quantity of blood might have been loſt; but is it not fair to expect, in ſuch a caſe, that this blood muſt, more or leſs, have been mixed with purulent matter, and particularly at the commencement of the hæmorrhage? This, however, I could not, by the ſtricteſt examination, diſcover to be the caſe; all, ſeemingly, being a tough mucus, that was coughed up. Hectic is, in general, a conſtant attendant in vomicas, varying in degree according to the progreſs ſuch collections of matter may have made; though, at the ſame time, I am well aware, that ſuch morbid appearances have frequently been diſcovered in the lungs after death, when no ſymptoms of hectic had ever before been evident;—no ſymptoms of hectic, as far as I can learn, had ever troubled our patient. By the term *vomica*, here made uſe of, I conſider it in the ſame light with the late Dr. Cullen, "Vomicam vocamus, quam vomicam *clauſam* appellant medici." According to Linnæus and Vogel, "Characteres vomicæ in *vomicam apertam* tantùm reſpiciunt."

But though, in our own minds, we may be able ſatisfactorily to account for this great diſcharge of blood, yet we derive no help from this ſource, in inveſtigating the cauſes of ſome more important ſymptoms, &c. The peculiar tightneſs and conſtriction of the cheſt, the acute pain extending from between the ſcapulæ to the cartilago xiphois, and the ſharp intolerable pain of the left arm.

From the preſcience of the laſt mentioned ſymptoms, I was too well aware of the imminent danger our patient was in, and had but too much reaſon to ſuſpect that his complaint, which hitherto ſeems generally to have baffled all medical aſſiſtance, was the ſame ſo properly and judiciouſly deſcribed by Dr. Heberden, in the ſecond volume of the Medical Tranſactions. Two very principal circumſtances, however, ſo unuſually met with with in cases of this nature, caused me to waver a little in my opinion: I could not by any means satisfy myself, when I considered the very early period of life at which this complaint made its appearance, and the uncommonly full and regular pulsation of the heart and arteries, that this was a case of *Angina Pectoris*. It has properly enough been observed by the late Dr. Fothergill, that as there are no two human bodies precisely alike, so there will be a great diversity of symptoms; the deviation, however, in this case, from the general progress of the disease (if it be considered as the angina pectoris) is such as to render it one of the most singular upon record. In the 5th volume of Medical Observations and Enquiries, a case of Angina is related by Dr. Fothergill, where his patient had only attained his 30th year, and which was the youngest subject he had ever seen affected with this disorder. In the case under discussion, however, it made its appearance at the early age of 21. —The general time of aggression is about the 50th year. Senac, in his excellent treatise "De Coeur," makes mention of several species of palpitatio, and, amongst the rest, Palp. a corde ossificato et Palp. ex aortæ angustiâ; not one, however, amongst the whole, corresponds in the least with the disease in question; nor have I been able to discover any thing similar to it, from consulting authors who have treated upon the subject.

Upon the whole, I am led to consider this as a singular and uncommon case, produced by no known cause, and terminated through the agency of circumstances, which perhaps dissection itself could not clearly have demonstrated. I am,

Gentlemen,

With the greatest respect,

Your's, &c. &c.

*East Retford,*

*March* 20, 1800.

THOMAS HULL.

*P. S.* In the last Volume of Duncan's Annals of Medicine, I inserted the case of a singular variety of Chorea Sancti Viti, (misprinted *Hall* for *Hull)*. As a considerable time has elapsed since I transmitted the case, and as some of your readers may be anxious to know the result arising from the continued use of the argent. nitrat. I here take the opportunity to inform them, that the patient has never suffered the least return of her fits since the time there specified.

